# Comparison Between the Facial Flow Lines of Androids and Humans

**DOI:** 10.3389/frobt.2021.540193

**Published:** 2021-03-22

**Authors:** Hisashi Ishihara, Saneyuki Iwanaga, Minoru Asada

**Affiliations:** ^1^ Graduate School of Engineering, Osaka University, Suita, Japan; ^2^ JST Sakigake/Presto, Tokyo, Japan; ^3^ Symbiotic Intelligent Systems Research Center, Institute for Open and Transdisciplinary Research Initiatives, Osaka University, Suita, Japan

**Keywords:** android robot, facial expression, displacement, human face, flow lines, Affetto, motion capture system analysis

## Abstract

The behavior of an android robot face is difficult to predict because of the complicated interactions between many and various attributes (size, weight, and shape) of system components. Therefore, the system behavior should be analyzed after these components are assembled to improve their performance. In this study, the three-dimensional displacement distributions for the facial surfaces of two android robots were measured for the analysis. The faces of three adult males were also analyzed for comparison. The visualized displacement distributions indicated that the androids lacked two main deformation features observed in the human upper face: curved flow lines and surface undulation, where the upstream areas of the flow lines elevate. These features potentially characterize the human-likeness. These findings suggest that innovative composite motion mechanisms to control both the flow lines and surface undulations are required to develop advanced androids capable of exhibiting more realistic facial expressions. Our comparative approach between androids and humans will improve androids’ impressions in future real-life application scenes, e.g., receptionists in hotels and banks, and clerks in shops.

## 1 Introduction

Advanced artificial systems are difficult to design because the numerous components have complex interactions with each other. System design is more difficult when the system components include uncertain properties. One such system is the face of an android robot. Android robots are humanoid robots with a soft surface for communication, especially on their face. The surface deformations result from the complex interactions between several face system components such as the soft skin sheet, skull-shaped shell to support the skin, transmission lines, and actuators. Tadesse and Priya (2012) pointed out that there can be several cases of mechanical friction between the skin and shell, and skin properties (e.g., the thickness, material, and elasticity) affect the overall movement. These properties are difficult to identify and tune during the design stage because they may change during the fabrication process (Ishihara et al., 2018).

Therefore, face system behavior (i.e., facial surface deformations) needs to be numerically analyzed after the components are assembled to improve the performances. The surface deformations of androids should be compared with those of humans because the former are replicating the latter. If android designers have sufficient knowledge on the differences between the surface deformations of androids and humans, they can infer why their androids give humans a strange impression and determine what materials and design technologies are required to improve the performance. Because this has been difficult with conventional, subjective evaluation methods (e.g., Kobayashi et al., 2003; Berns and Hirth, 2006; Hanson, 2006; Hashimoto et al., 2006; Macdorman and Ishiguro, 2006; Allison et al., 2009; Bartneck et al., 2009; Becker-Asano and Ishiguro, 2011; Lin et al., 2011; Baldrighi et al., 2014; Lazzeri et al., 2015), there have neither been objective design guidelines for human-like android robots, nor effective design policies for advanced android robots. Instead, android faces have been designed from the intuitions and experiences of their creators through trial-and-error.

Several studies have attempted to measure the facial deformations of androids and compare them with humans. Hashimoto and Yokogawa (2006) and Hashimoto et al. (2008) measured the two-dimensional displacements of seven facial feature points with geometrically apparent locations (e.g., the corners of the eyes and mouth) when a human female and her replica android attempted to show six basic emotions (i.e., anger, disgust, fear, happiness, sadness, and surprise). The displacements of these feature points were obtained from video images and compared to verify if the android could replicate the facial expressions successfully. Yu et al. (2014) investigated these basic emotions by comparing 13 facial feature points for a human male and his replica android with an optical motion capture system and calculated the average difference between the three-dimensional displacements as a similarity index of their facial deformations. The above studies analyzed the facial deformations as sparse distributions of displacement vectors. However, Cheng et al. (2013) pointed out the importance of analyzing dense distributions of displacement vectors for the entire facial surface to aid the mechanical design of androids. They measured the displacements of approximately 200 facial lattice points when a human male and female android attempted to show four typical emotional expressions (i.e., happiness, anger, sadness, and shock). Then, they analyzed how the displacement distribution patterns differed and used the results to change the design of the actuation mechanisms of the android. They successfully improved the similarity of the displacement distributions and the human-like impression of the android.

Although the above comparative analyses seem a promising approach for evaluating androids, they only focused on a limited number of typical emotional expressions. These typical expressions are only a part of the rich and various patterns of facial motions that are a complex combination of several independent motions. Thus, it is intrinsically difficult to characterize human-specific deformations and investigate independent motions produced by individual actuators of androids.

Ishihara et al. (2017) previously pointed out that the displacements for the independent facial motions of humans need to be measured in detail. Action units (AUs) are independent facial motions such as rising the inner brow and wrinkling the nose that have been defined in the Facial Action Coding System (FACS) (Ekman et al., 2002). FACS exhaustively defines a set of independent AUs and explains that every emotional and non-emotional facial expression can be decomposed into one or multiple AUs. Ishihara et al. (2017) measured the dense displacement distributions when a human male shows each AU around the mouth, and found that the human face is characterized by moving mostly along a single direction for each facial point for various AUs. Ishihara et al. (2018) also measured the dense displacement distributions for each independent facial motion that is produced by a single actuator of an android (hereafter called a deformation unit (DU)). They found that the time sequences of displacement distributions can be approximated by sigmoid functions, which implies that it can be used with a feedforward controller to improve the control precision for the time sequence of a facial surface deformation.

The previous two studies (Ishihara et al., 2017; Ishihara et al., 2018) showed that the deformations for each AU and DU need to be measured in detail to obtain new insight into the design of facial motions. However, such deformations have not been compared between androids and humans. In this study, we measured the displacement distributions of each AU and DU for three adult males and two androids, and compared the distribution patterns in terms of their flow lines and surface undulations. The main purpose of this study is to reveal differences between humans and androids. Therefore, in this study, we do not regard the age and gender differences within humans and androids to be an issue, as we assume that the differences within humans or androids are much smaller compared to the differences than between the two group. For a fair comparison, the size and shape of the faces were normalized among female/child androids and humans.

## 2 Methodology

### 2.1 Robots and Human Participants

We investigated the facial motions of two android robots and three adult males. One of the androids was a female adult android (A-lab Co., Ltd., A-lab Female Android Standard Model). It had nine effective actuators to move its facial skin. Table 1 describes its DUs produced by the nine actuators. The other android was a child android named Affetto (Ishihara et al., 2011; Ishihara and Asada, 2015; Ishihara et al., 2018). It had 16 actuators for moving its facial skin, and Table 2 describes its DUs. Each actuator was a pneumatic linear cylinder or rotary bane actuator installed in the head, and their target positions could be set as one-byte positional commands from 0 to 255. As the command increased, the face moved as described in Tables 1, 2. For example, the upper eyelid of the female android was at its highest position when DU1 was set to 0 and lowest when it was set to 255. Only three actuators in the female android (1, 2, and 12) and Affetto (1, 2, and 9) had potentiometers at their output axis for feedback control. Although these two android robots differed in size and appearance, they had a similar facial structure and mechanism designed and manufactured by the same company (A-lab Co., Ltd.).

**TABLE 1:** Actuator numbers and deformation units of the female android.

Number	DU description
1	Lowering the upper eyelid
2	Turning up the eye pit
3	Raising the lower eyelid
4	Raising the outer eyebrow
5	Raising the inner eyebrow
6	Raising the cheek
7	Centering the upper lip
8	Pulling the corner of the mouth
9	Opening the jaw

**TABLE 2:** Actuator numbers and deformation units of Affetto.

Number	Description
1	Lowering the upper eyelid
2	Turning up the eyepit
3	Raising the lower eyelid
4	Raising an eyebrow
5	Centering the eyebrow
6	Raising the middle cheek
7	Pulling the middle cheek to the side
8	Pulling the lower cheek to the side
9	Lowering the corner of the mouth
10	Centering the upper lip
11	Centering the lower lip
12	Opening the jaw
13	Lowering the nose
14	Pulling the corner of the mouth
15	Lowering the lower lip
16	Raising the corner of the mouth

The three Japanese adult males (mean age = 22.7 years, SD = 0.2 years) who participated in this study were students at Osaka University in Japan. They were asked to show each of the 44 AUs defined in FACS, which are shown in Table 3. These three participants practiced showing AUs with a mirror until they were satisfied. The three participants were unaware of the facial expressions specified by FACS (i.e., there was no assurance that the measured motions would match the motions defined in the FACS). For example, the same AU could be shown as different motions, and similar motions could be observed as different AUs. Such imperfectness by the three adult males was allowed because our aim was not to visualize the “perfect” displacement distributions for each AU but to reveal the “human-like” characteristics of facial motions expressed by ordinary people.

**TABLE 3:** Action units of humans.

Number	AU description	Number	AU description
1	Inner brow raiser	24	Lip pressor
2	Outer brow raiser	25	Lip part
4	Brow lowerer	26	Jaw drop
5	Upper lid raiser	27	Mouth stretch
6	Cheek raiser	28	Lips suck
7	Lid tightener	29	Jaw thrust
8	Lips toward each other	30	Jaw sideways
9	Nose wrinkler	31	Jaw clencher
10	Upper lip raiser	32	Lip bite
11	Nasolabial furrow deepener	33	Cheek blow
12	Lip corner puller	34	Cheek puff
13	Cheek puffer	35	Cheek suck
14	Dimpler	36	Tongue bulge
15	Lip corner depressor	37	Lip wipe
16	Lower lip depressor	38	Nostril dilator
17	Chin raiser	39	Nostril compressor
18	Lip puckerer	41	Lid droop
19	Tongue out	42	Slit
20	Lip stretcher	43	Eyes closed
21	Neck tightener	44	Squint
22	Lip funneler	45	Blink
23	Lip tightener	46	Wink

### 2.2 Measurement

Facial motions were measured as three-dimensional displacement vectors distributed on the face. An optical motion capture system with six infrared cameras (OptiTrack Flex13) was utilized to capture the movements of hemispherical infrared reflection markers with a 3 mm diameter attached to the right halves of the facial skin of the female android, Affetto, and the three adult males. The frame rate was 120 frames per second. For Affetto, we utilized the data of the displacement vectors obtained in the previous study (Ishihara et al., 2018).

**FIGURE 1:**
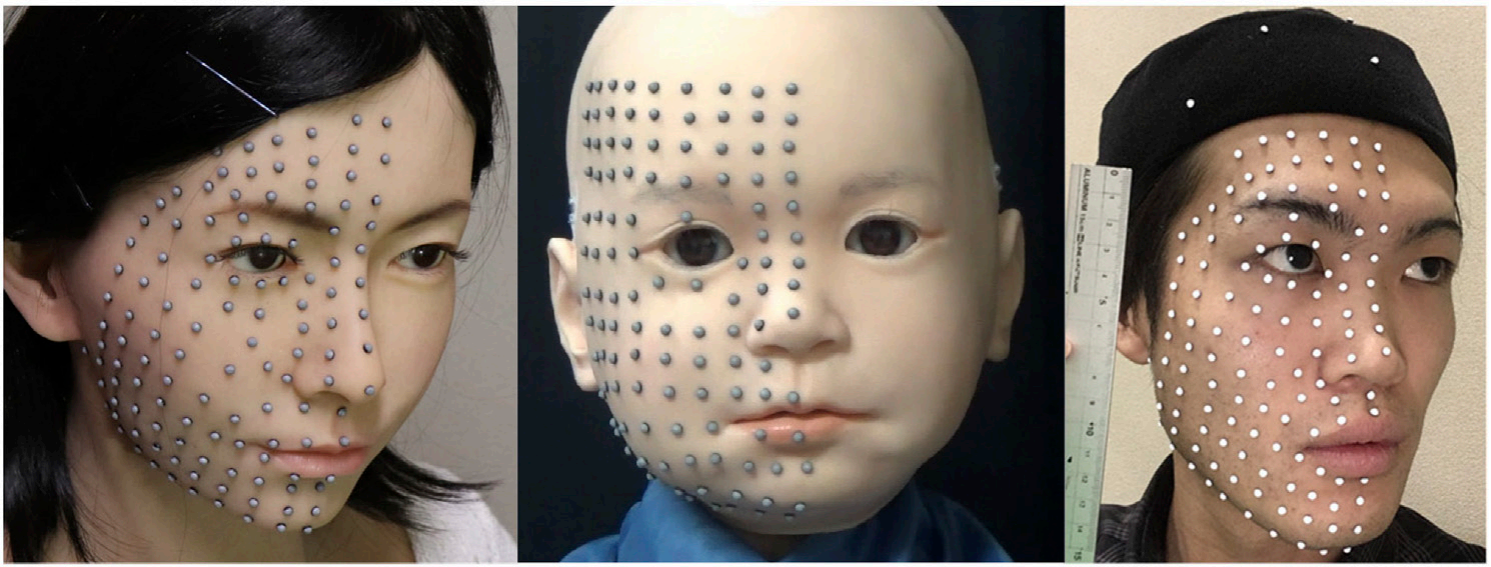
Marker locations on the neutral faces of the female android **(left)**, Affetto **(middle)**, and one of the adult males **(right)**.

**FIGURE 2:**
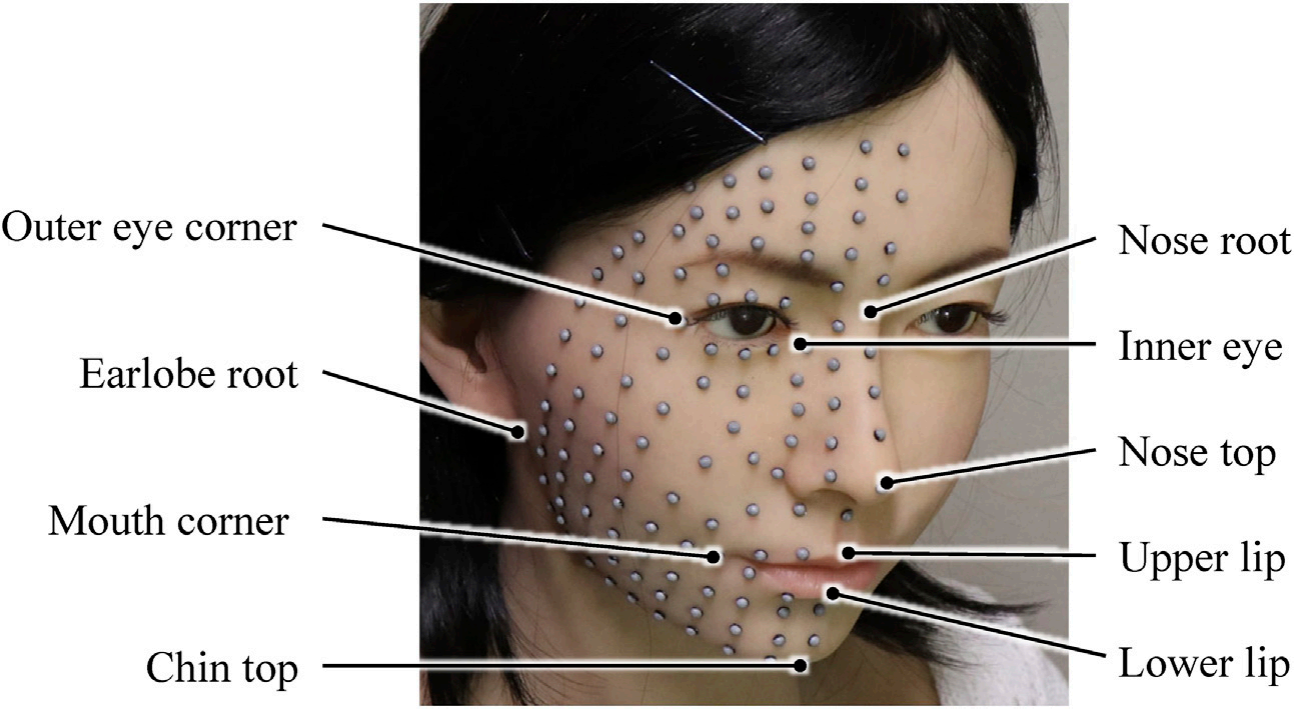
Locations of nine reference markers on the female android that were used to compensate for facial shape differences among the androids and adult males.

Figure 1 shows the marker locations on the neutral faces of the female android, Affetto, and one of the adult males. In total, 120 and 116 markers were attached to the faces of the female android and Affetto, respectively, at intervals of approximately 10 mm. We attached 125, 103, and 117 markers to each of the three adult males. To calibrate and normalize the shape difference of the faces, we selected nine representative points as reference markers, as shown in Figure 2: the nose root, outer and inner corners of the eyes, top of the nose, earlobe root, corners of the mouth, tops of the upper and lower lips, and top of the chin.

The nine DUs of the female android and 16 DUs of Affetto were measured one by one. First, the positional command was set to 0 for one of the actuators so that the initial marker positions could be measured. It was then changed to 255 so that the final marker positions could be measured. When one DU was measured, the positional commands for the other actuators were set to the values for displaying a neutral face, as shown in Figure 1.

The facial movements for the 44 AUs of the human participants were also measured one by one. First, the participants practiced one of the AUs by watching their facial movements in a mirror. Next, they presented their neutral faces so that the initial marker positions could be measured. They then showed the AU so that the final marker positions could be measured. The distributions of the displacement vectors for the DUs and AUs were calculated as the three-dimensional positional differences between the initial and final marker positions.

Note that the AUs and DUs are not precisely compatible. A DU is an exact unit of artificial facial motion produced by a single actuator of the android. On the other hand, an AU is a superficial unit of human facial action subdivided and extracted from complex facial expressions. This means that an AU can be replicated more precisely by any DU combinations than a single DU. However, we do not deal with the DU combinations because our focus is not on the best replication performance but the elemental features of facial motions in this study.

A coordinate system was defined based on the initial positions of several reference markers. The top of the chin was set as the origin, and the *y*-axis was defined as the direction from the origin to the nose root. The direction perpendicular to the *y*-axis from the nose top was defined as the direction of the *z*-axis. Based on the *y*- and *z*-axes, the *x*-axis was automatically defined for a left-handed orthogonal coordinate system.

### 2.3 Analysis Method

To compare the facial movements among the female android, Affetto, and the three adult males, two types of data preprocessings were conducted on the obtained three-dimensional displacement vectors. The first one was to compensate for differences in the facial shape. The initial and final marker positions of the female android, Affetto, and the adult males were transformed with thin plate spline warping (Duchon, 1977) (i.e., non-linear smooth transformation of multivariate data) so that the initial positions of the nine reference points matched. After this transformation, the displacement vector positions could be compared between different faces. The second one was data interpolation to improve the spatial resolution. Natural neighbor interpolation (Sibson, 1981) was performed on the measured displacement vectors in order to calculate lattice point data at intervals of 1 mm on the *x*–*y* plane. The interpolated vectors were averaged among the three adult males for visualization and analysis. The interpolated vectors were averaged for five and ten measurements of the female android and Affetto, respectively, with MATLAB R2019a.

After the preprocessing, the distributions of the displacement vectors for the androids and adult males were compared. Flow lines (i.e., global trends of displacement vectors) were observed from the vector maps on the *x*–*y* plane (i.e., frontal face view), while the surface undulations were observed from the distributions of the *z* component for the displacement vectors on the *x*–*y* plane. In other words, we regarded the *z* component as the index of surface undulations because the faces were almost convex. Specifically, we regarded the positive and negative *z* components as the skin elevations and depressions, respectively.

The complexity of the flow lines was calculated as the standard deviation of the displacement vector orientations around the peak of the maximum displacement for each motion. Thus, the complexity $C_{r}$ of a motion within an area with the radius of *r* was calculated as

$$C_{r}=\sqrt{\frac{1}{N_{r}}\underset{d_{i}<r}{\sum }{\left(\theta_{i}-\bar{\theta }\right)}^{2}},$$

where $N_{r}$ is the number of the displacement vector $v_{i}$ whose distance $d_{i}$ from the peak point is less than *r*, $\theta_{i}$ is the angle between the displacement vector $v_{i}$ and the *y*-axis, and $\bar{\theta }$ is the average of $\theta_{i}$. Vectors smaller than 0.2 mm were ignored in this analysis.

## 3 Results

In this section, we introduce the representative distributions of the displacement vectors in the eye, forehead, and mouth areas. Nine types of DUs and AUs were chosen to compare the flow lines and surface undulations of the androids and adult males. Next, the distributions of the peak points of the maximum displacement length for each DU and AU were evaluated to classify the motions based on the positions of the peak points. Finally, the complexity index values of the androids and adult males were compared for each classified group of DUs and AUs.

### 3.1 Displacement Vector Maps

#### 3.1.1 Eye Area

**FIGURE 3:**
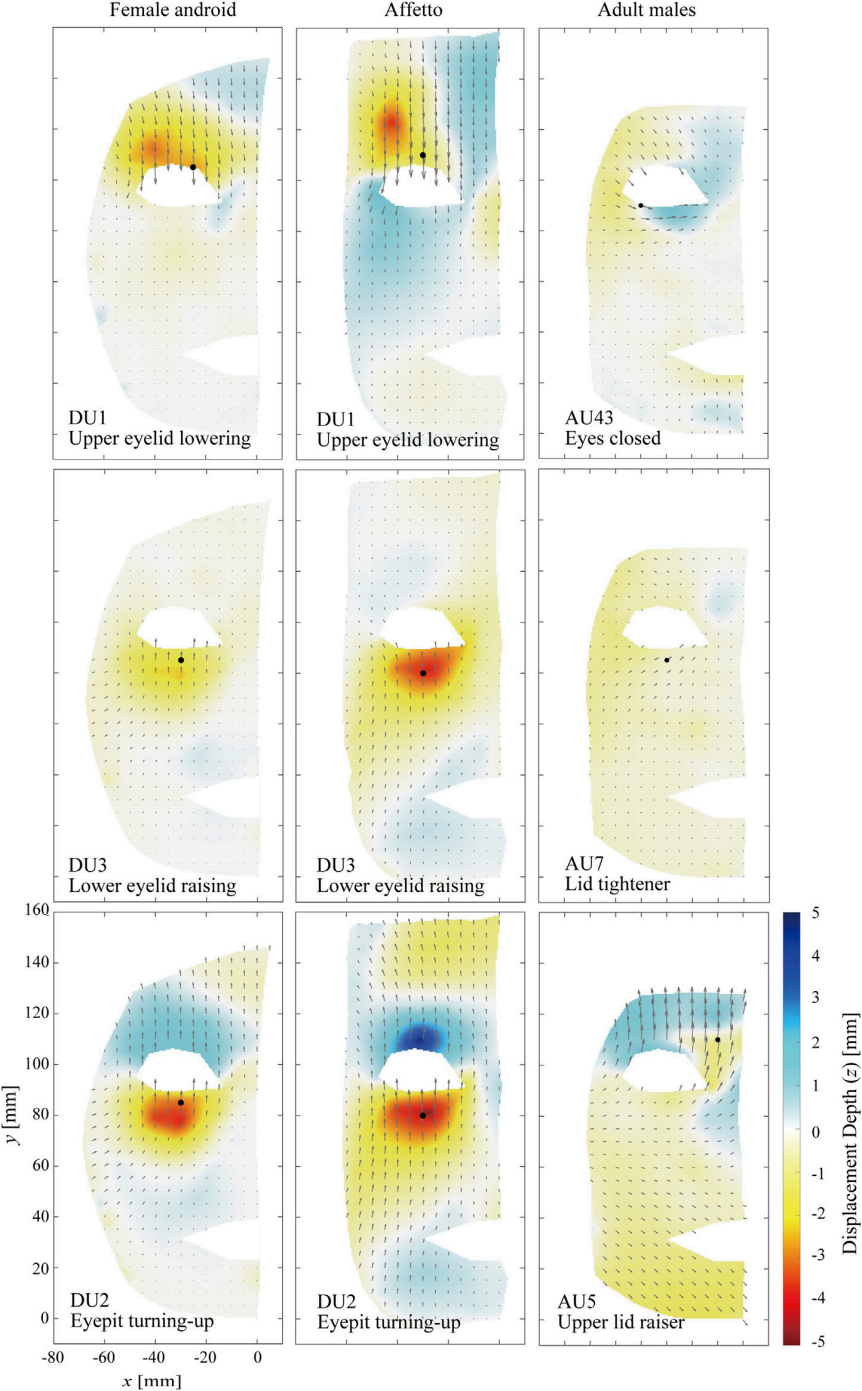
Displacement distributions around the eye. The motions to raise the upper eyelid **(top)**, raise the lower eyelid **(middle)**, and to look up **(bottom)** are depicted.

Figure 3 compares the distributions of the displacement vectors for three types of facial motions around the eyes on the *x*–*y* plane. The subfigures of the left, middle, and right columns correspond to the female android, Affetto, and the average of the three adult males, respectively. Each row shows one of the DUs and the closest motion corresponding to an AU. The motions to lower the upper eyelid, raise the lower eyelid, and look up are depicted in the top, middle, and bottom rows, respectively. The orientations and lengths of the black arrows represent the orientations and amplitudes of the displacement vectors at each point. The heat maps represent the *z* component of the displacement vectors. Blue regions indicate depressed areas, whereas yellow and red regions indicate elevated areas. The black dots represent the peak point with the maximum displacement length.

The displacements were distributed across the entire face for both the androids and adult males, including the top of their foreheads and skin around their mouths. In addition, there were apparent borders between elevated and depressed areas. The distribution patterns differed for the androids and adult males. The flow lines were almost straight and vertical for the androids. In contrast, for the adult males they were diagonal around the upper eyelid and horizontal around the lower eyelid for AU43 of the adult males and diagonal at the lower eyelid for AU7 and AU5. Furthermore, the displacement lengths gradually decreased with increasing distance from the point of the maximum displacement length in the androids, whereas the lengths increased around the mouths of the adult males (AU43 and AU7).

#### 3.1.2 Forehead Area

**FIGURE 4:**
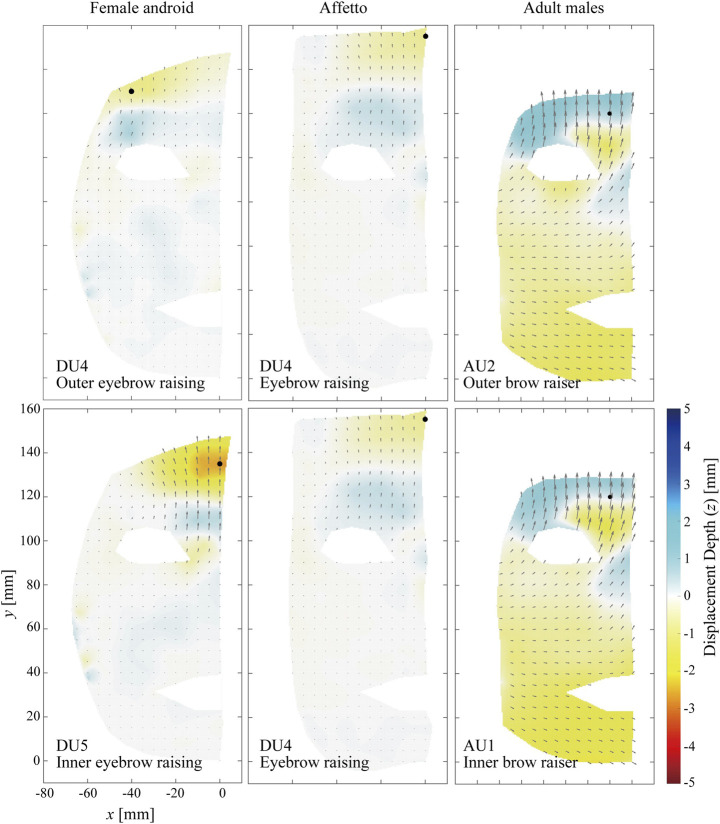
Displacement distribution around the forehead. The motions to raise the outer eyebrow **(top)** and to raise the inner eyebrow **(bottom)** are depicted.

Figure 4 compares the distributions of the displacement vectors for two types of facial motions around the forehead on the *x*–*y* plane. Because Affetto had only one actuator for the eyebrows, the same DU was adopted for this comparison. The motions to raise the outer eyebrow and raise the inner eyebrow are depicted in the top and bottom rows, respectively.

The flow lines differed between the androids and adult males similar to the eye area, especially toward the center of the forehead. They were vertical in the androids (especially DU5 for the female android) but diagonal in the adult males (AU1 and AU2). Furthermore, a horizontal color border was observed between the depressed and elevated areas around y=120 for both the androids and adult males, but the color distributions were opposite. The top of the forehead (approximately y>120) was yellow (elevated), while the bottom of the forehead (approximately y<120) was blue (depressed) for the androids. In contrast, the top of the forehead was blue while the bottom of the forehead was yellow for the adult males. The displacement lengths also increased around the mouths of the adult males, similar to the eye area.

#### 3.1.3 Mouth Area

**FIGURE 5:**
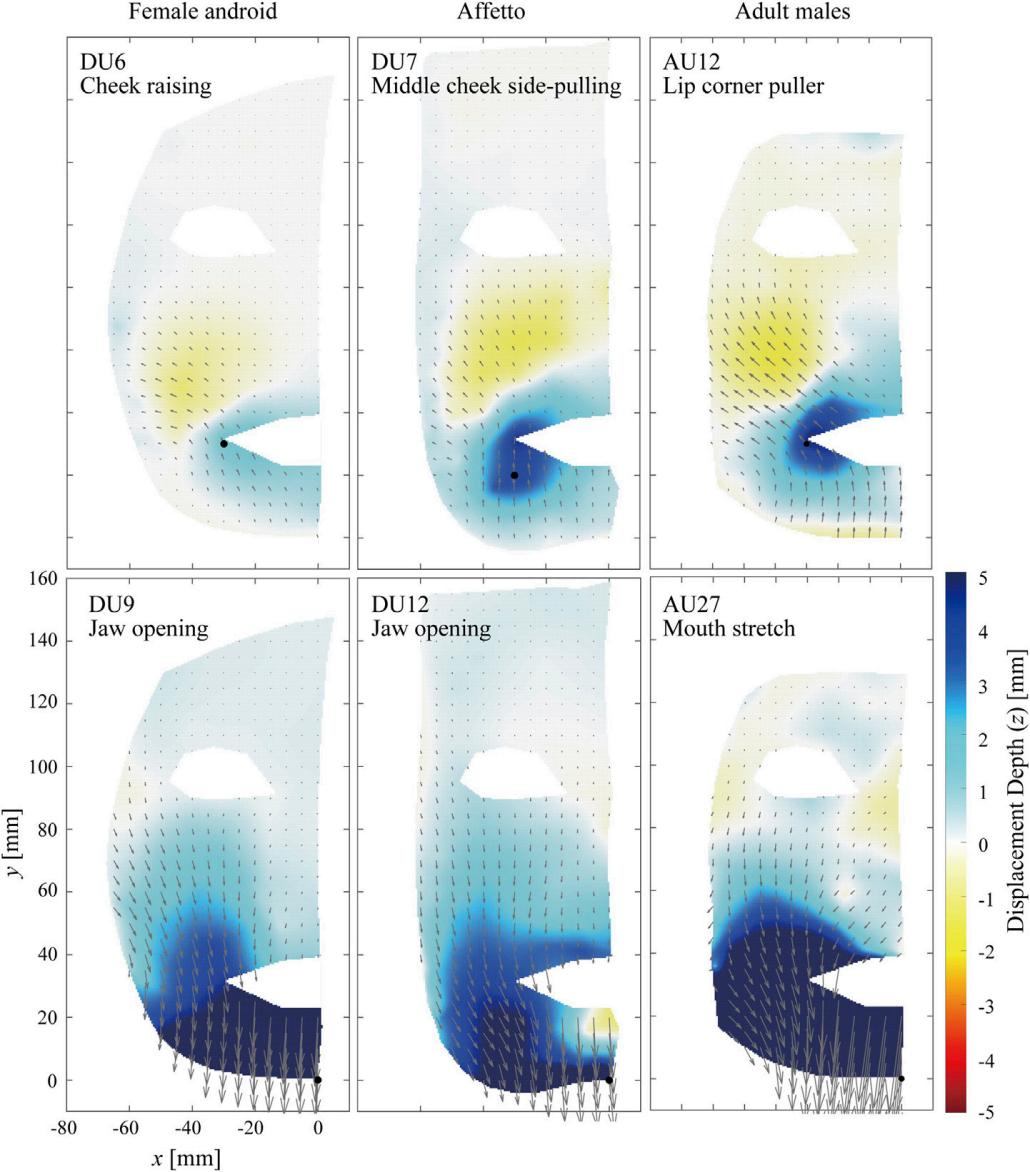
Displacement distribution around the mouth. The motions to raise the corner of the mouth **(top)** and to open the jaw **(bottom)** are depicted.

**FIGURE 6:**
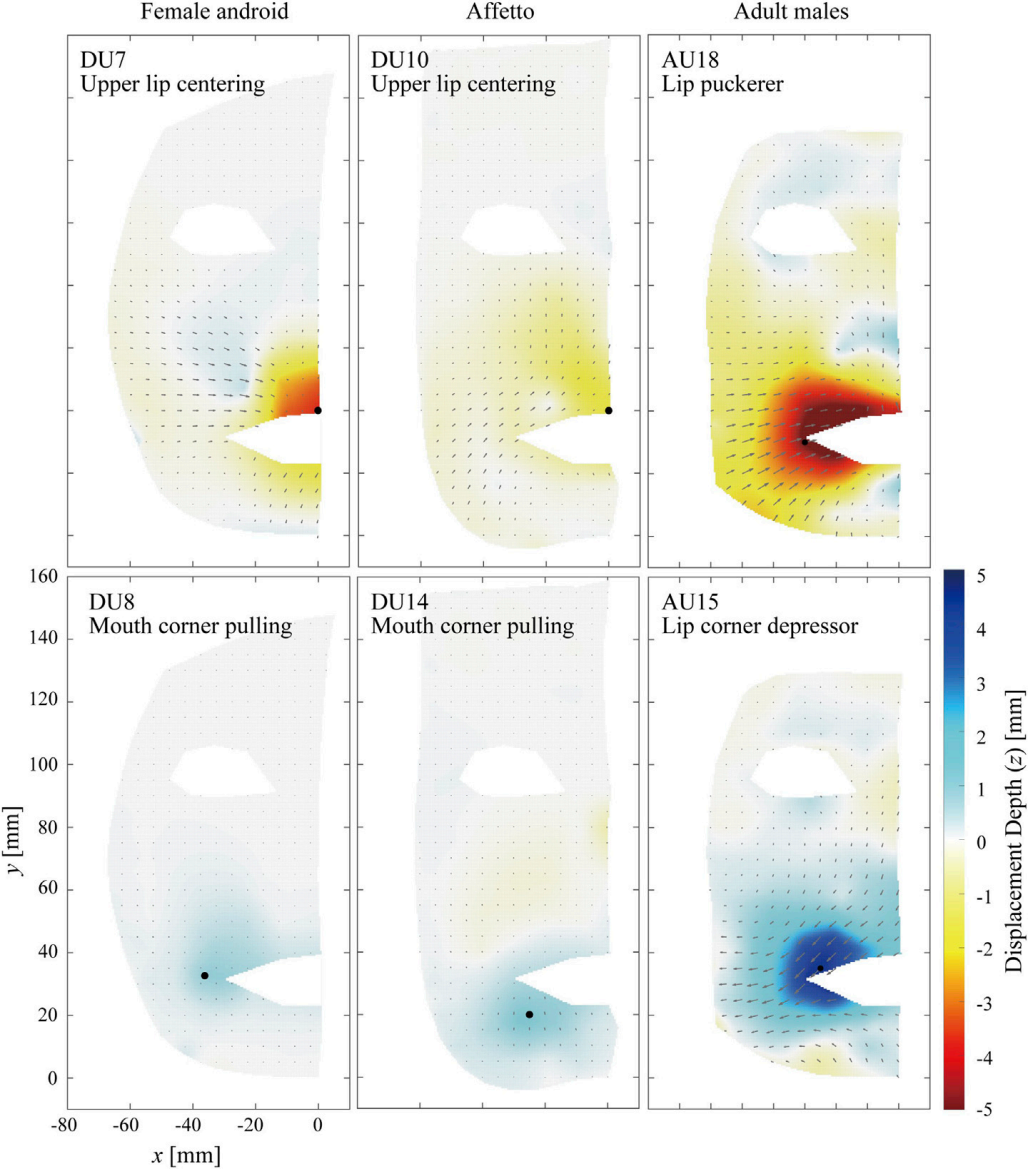
Displacement distribution around the mouth. The motions to protrude the lip **(top)** and to pull the corner of the mouth to the side **(bottom)** are depicted.

Figures 5 and 6 compare the distributions of the displacement vectors for four types of facial motions around the mouth on the *x*–*y* plane. The flow lines and color distributions for the androids and adult males are similar in Figure 5, where the top and bottom rows are the motions to raise the corner of the mouth and to open the jaw, respectively. For the former motion, the skin was depressed around the mouth while it was elevated around the cheek for both the androids and adult males. For the latter motion, the skin of the lower face was entirely depressed by the motion of the jaw.

In contrast, Figure 6 shows differences between the androids and adult males, where the top and bottom rows show the motions to protrude the upper lip and pull the corner of the mouth to the side, respectively. For example, apparent flow lines in the cheek area were oriented to the right bottom for DU7 of the female android, whereas they were on the outer side of the jaw and oriented to the right top for AU18 of the adult males.

### 3.2 Locations of Peak Points

**FIGURE 7:**
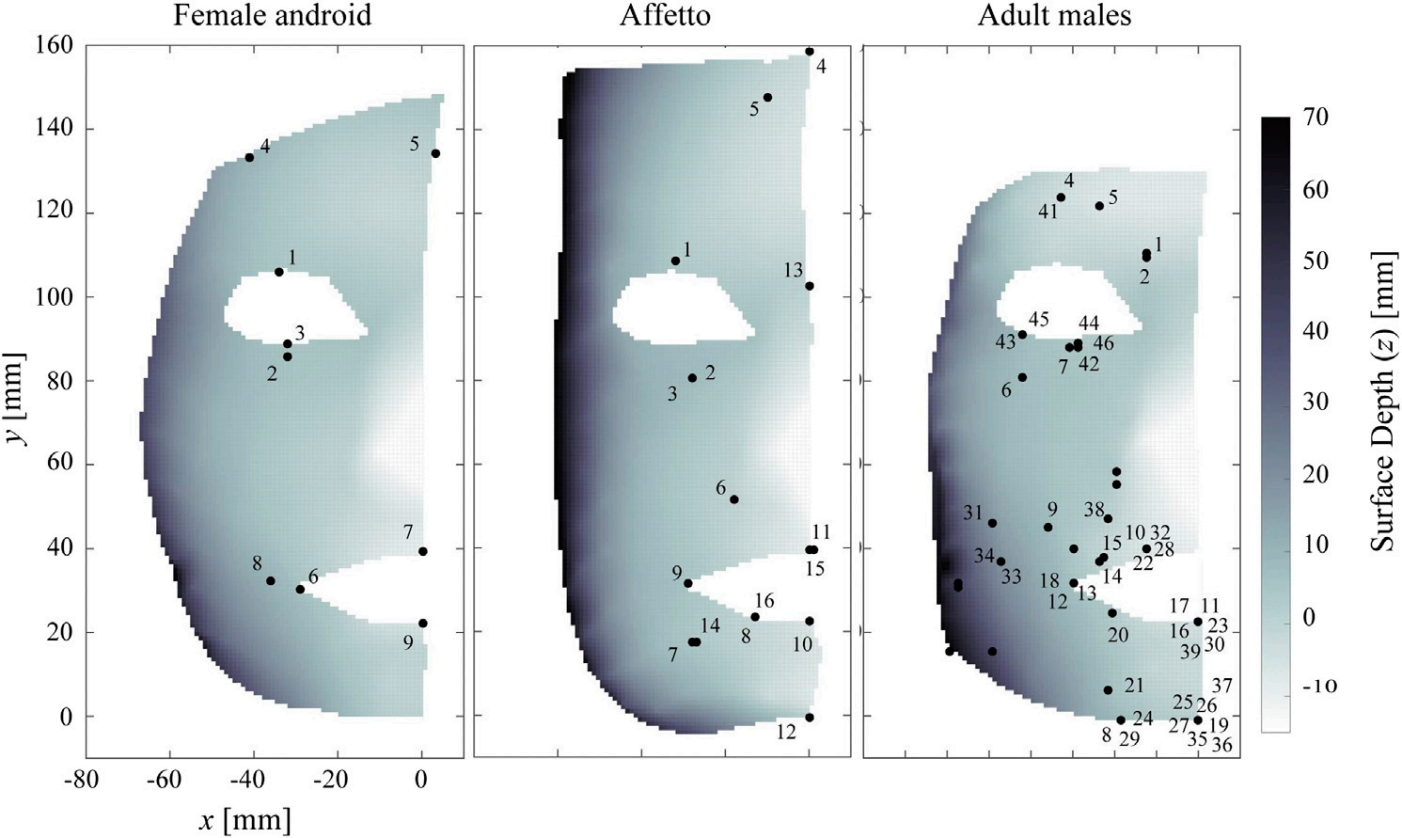
Locations of peak points in the androids and adult males. Blank areas without peak points can be observed in the middle at y=60 to y=80.

Figure 7 shows the locations of every peak point. The peak points were distributed across the entire face, although there seemed to be a blank strip around the upper cheek (i.e., y=60 to y=80). Therefore, we divided the motions into two groups based on the locations of the peak points: the upper face motions and lower face motions.

The upper face motions of the female android were DU1–5, while those of Affetto were DU1–5 and 13. The upper face motions of the adult males were AU 1, 2, 4–7, and 41–46. The rest of the DUs and AUs were regarded as lower face motions.

### 3.3 Complexity

**FIGURE 8:**
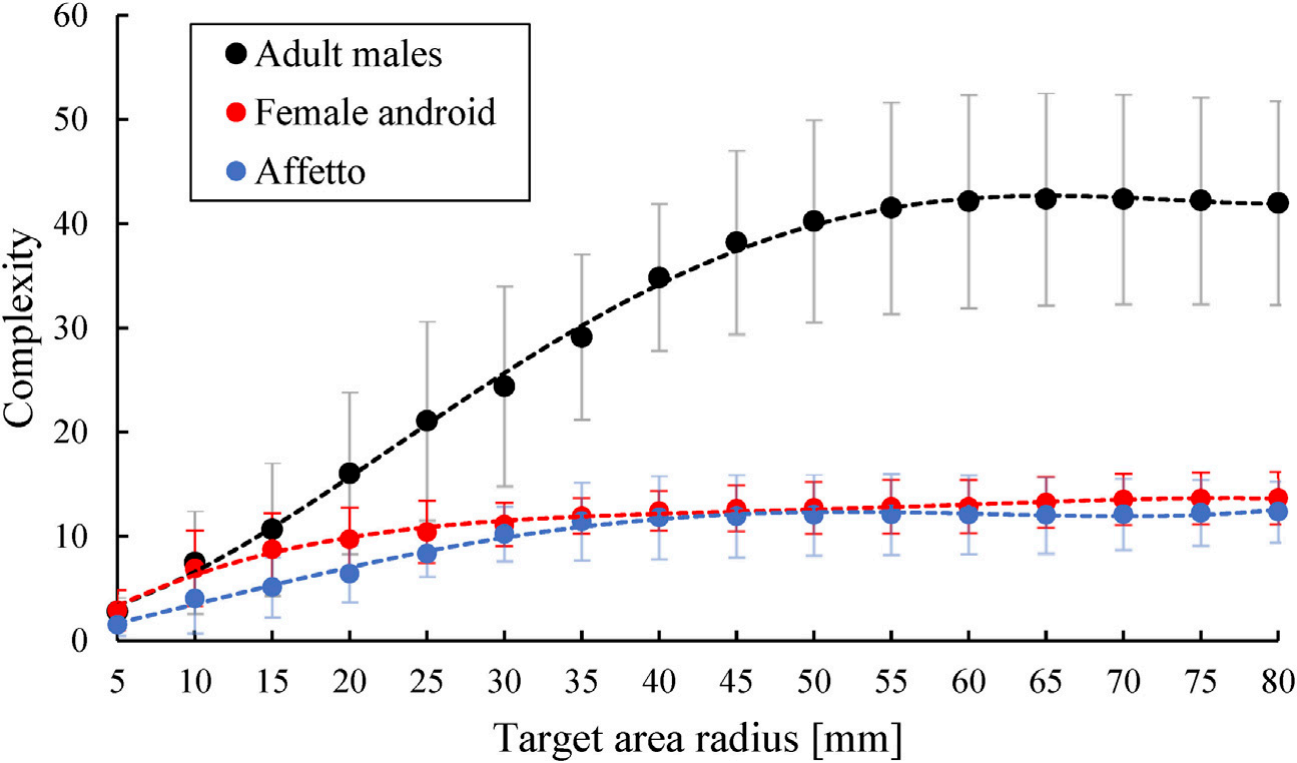
Complexity of the upper face motions for different target area radii.

**FIGURE 9:**
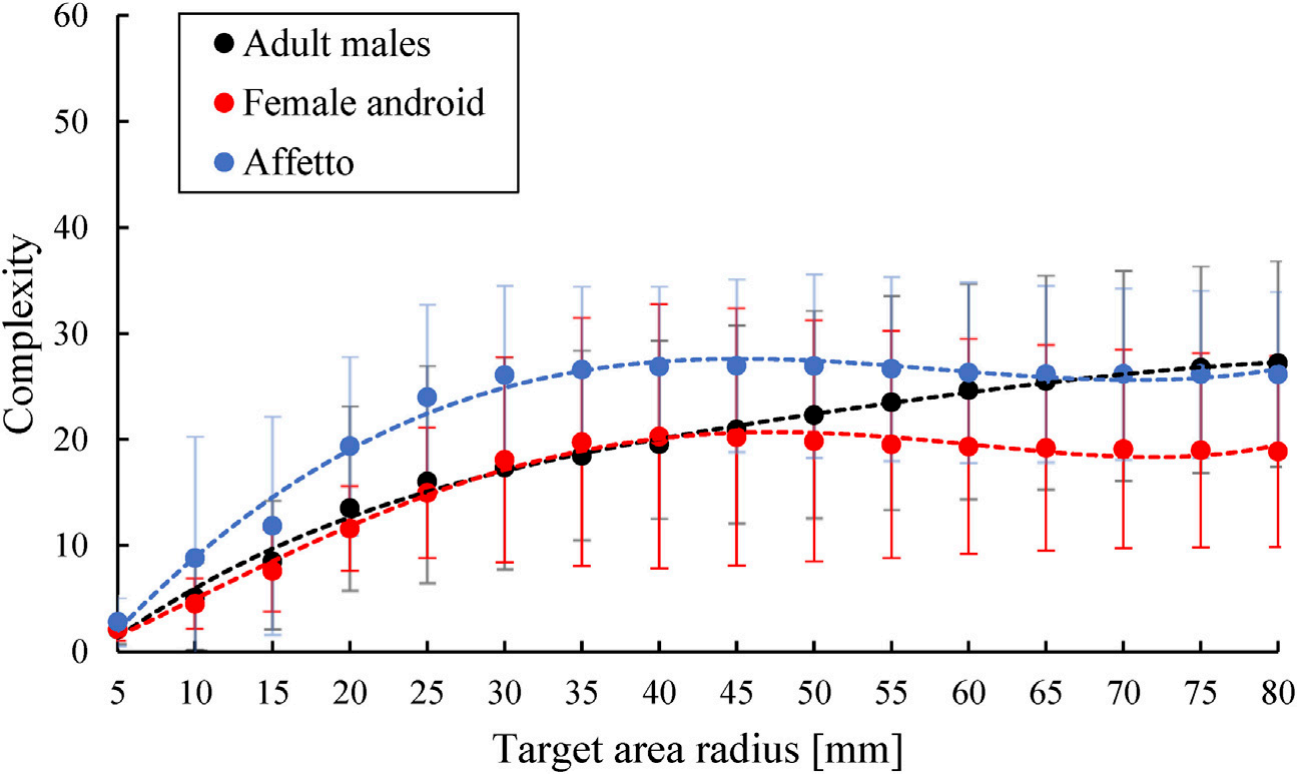
Complexity of the lower face motions for different target area radii.

Figures 8 and 9 compare the complexities of the upper and lower face motions, respectively, for the androids and adult males. The vertical axis indicates the complexity $C_{r}$, while the horizontal axis indicates the radius *r* of the target area. The average and standard deviation of $C_{r}$ for every upper or lower face motion were plotted with fourth-order approximation functions.

The androids and adult males showed a noticeable difference in the complexity of the upper face motions. The complexity was greater for the adult males when the radius was above 20 mm. In contrast, the androids and adult males showed similar levels of complexity for the lower face motions. Overall, the complexity was highest for the upper face of the adult males and lowest for the upper face of the androids.

## 4 Discussion

### 4.1 Difference in Flow Lines

The first difference between the androids and adult males was in their flow lines, especially the eye and forehead areas. The flow lines tended to be almost straight and vertical for the androids but were curved and non-vertical for the adult males, as shown in Figures 3 and 4. This was not because of the differences in facial shape because this was accounted for so that the geometric points would match in a three-dimensional space. This difference between the androids and adult males was supported by the complexity $C_{r}$. Figures 8 and 9 show that the flow lines were simplest in the upper face areas of the androids, while they were the most complex in the upper face areas of the adult males.

The androids’ simple flow lines were because their face mechanisms were simple: a limited skin area was actuated according to a simple trajectory, and the surrounding areas moved passively (e.g., up and down around the eyes). In contrast, the face mechanisms are more complex for humans: each muscle moves several skin points in different directions at the same time because the muscle shrinks and its surface is connected to the skin surface in several regions. For example, the orbicularis oculi muscle closes the eye by shrinking while one end is fixed to the inner corner of the eye. Meanwhile, the skin area is fully stuck to the surface of the muscle. Therefore, the displacement vectors orient toward the inner eye corner at each point of the skin around the eye (i.e., these vectors are in different directions in different positions). This hypothesized explanation appears reasonable, as the complexity in the upper face area of the adult males, where the muscles are more closely and extensively connected to the skin due to the lack of adipose tissue, was significantly higher than in the lower face area.

There are two possible reasons for the high complexity in the upper face of the adult males: the flow lines were curved as discussed above, and incidental motions were produced in the lower face when the adult males attempted to produce a motion only in the upper area. For example, Figures 3 and 4 show that the lower faces of the adult males also moved approximately by up to 2 mm, and the orientations were different for the upper and lower face areas. This unintentional compound motion may have contributed to the high complexity and differences between the androids and adult males.

Thus, humans’ facial flow lines were more complex than androids’ in the upper face areas. Although AUs and DUs are not precisely compatible, as noted in section 2.2, this fact is crucially important for android designers. This is because the precise replication of humans’ curved AUs can not be expected with a single unit of androids’ straight DUs. One possible solution for this mismatch is the adoption of combinations of DUs to replicate a single AU. Comparison of flow lines between AUs and combinations of several DUs is one of the future issues. Another solution is redesigning a face mechanism for a problematic DU so that the flow lines would match an AU. This redesigning includes not only the actuation force trajectory but also the skin sheet structure.

Additionally, the displacement distributions of the adult males were quite similar for AU1, AU2, and AU7, as shown in Figures 3 and 4. This means that the adult males could not show these motions in different ways with different muscles or motor commands despite trying to do so according to the descriptions of each AU in the FACS. In other words, the actual degrees of freedom available for the human face to show AUs are fewer than those defined in the FACS. This suggests that a “perfect” facial mechanism that can differentiate all AUs is unnecessary when replicating an average person’s features in an android robot. Comparing the degrees of freedom among humans of different ages, sexes, and experience with facial acting and training is one of our future topics of research for the design of android robots.

### 4.2 Difference in Surface Undulations

**FIGURE 10:**
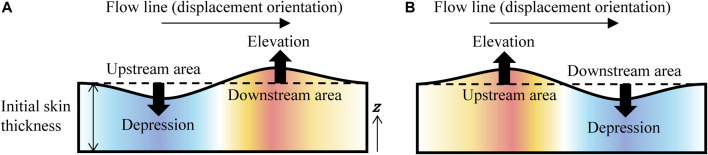
Relationships between a flow line and surface undulations in the **(A)** androids and **(B)** adult males.

The second difference between the androids and adult males was found in the skin surface undulation patterns around the upper face, especially the forehead motions shown in Figure 4. The skin tended to be depressed in the upstream areas of the flow lines and elevated in the downstream areas for the androids, as shown in Figure 10A. Inversely, they tended to be elevated in the upstream areas while depressed in the downstream areas for the adult males, as shown in Figure 10B.

**FIGURE 11:**
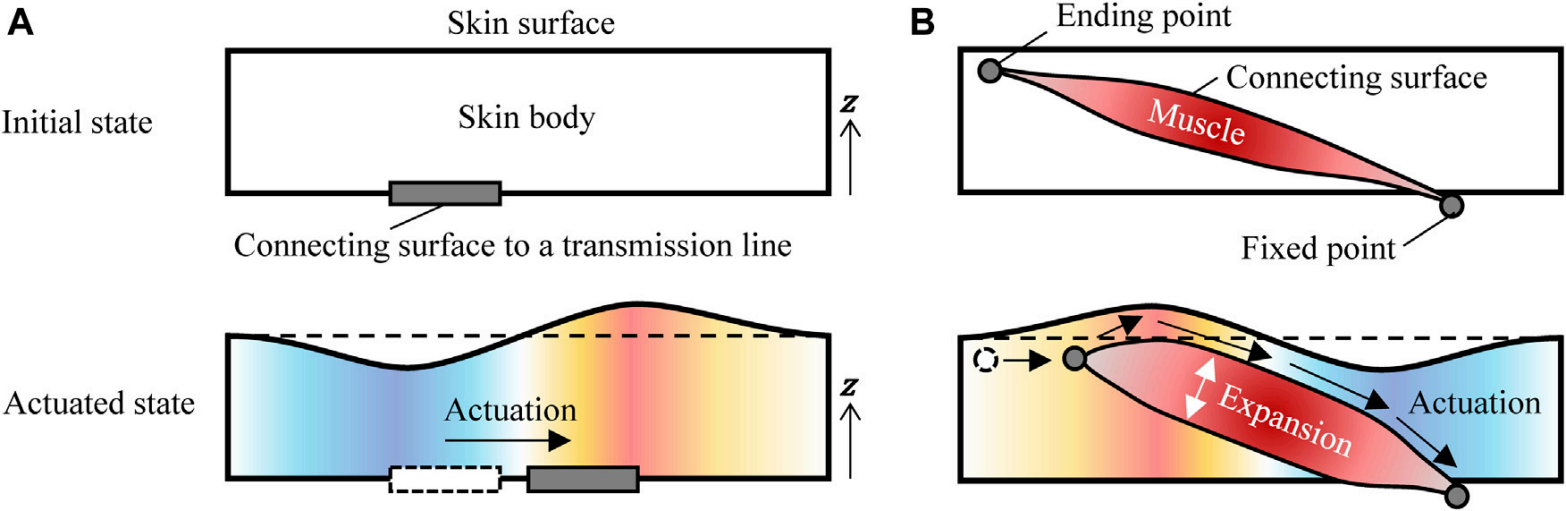
Possible mechanism for the surface undulations in the **(A)** androids and **(B)** adult males.

Because the flow lines reflect the transfer path of the skin material, the surface undulations of the androids were simply the result of the volume transfer from the upstream areas to the downstream areas produced by the skin movement of a surface connected to a transmission line, as shown in Figure 11A. However, the inverse surface undulations of the adult males cannot be explained with such a simple mechanism. Why does the downstream area seem to lose volume even though the skin material is flowing into this area? What happens in the downstream and upstream areas? One possible explanation is the combination of three features of the human skin system: 1) the human facial muscles expand themselves, 2) they connect the skin surface to the bone diagonally, and 3) the muscle surfaces are connected to the surrounding skin components. As shown in Figure 11B, this means that the surface can be elevated in the upstream areas (around the end point of a muscle near the skin surface) as muscles expand and depressed in the downstream areas (around the fixed point of a muscle to the bone) as muscles contract.

The above surface undulations in the adult males are quite challenging to replicate in androids. This is because the designers need to control the flow lines on the *x*–*y* plane and the undulations simultaneously. Flow line control can be achieved relatively easily by tuning the motion trajectories of the internal mechanisms, their combinations, and the stiffness of the skin materials. However, undulation control requires additional mechanisms to elevate and depress the skin surface at several areas unless muscle-like actuators are embedded in the skin. Innovative composite motion mechanisms are necessary to improve an androids’ replication of human facial motions.

### 4.3 Limitations

Because only three Japanese young adult males participated in this study, it is difficult to conclude that the identified features above are common in humans. There should be non-negligible differences in the faces of people when considering facial deformation mechanisms. For example, skin material properties such as the stiffness and surface tension change with age and physique. The power and controllability of facial muscles can also change with age and should be different between males and females or depending on one’s occupation and culture. Physical and mental conditions may also affect facial motions.

Therefore, there should not be only one set of motion characteristics for humans. Instead, there should be acceptable ranges of displacement distributions and motion characteristics for human facial motions, and their subtle differences should express different personalities. Further measurements and investigations are necessary with more participants having various backgrounds to determine the acceptable ranges for android design and to establish methods for designing androids with different personalities.

Because we measured the facial motions in an artificial scenario in which human participants presented a single AU, it is difficult to state the extent to which the found characteristics are expressed in real life. Experiments in more real-life scenarios are required in future works.

Because the two androids used in this study had similar mechanical structures, the displacement distributions of each DU were almost identical. However, the displacement distribution can be different for other androids with different mechanical structures (e.g., Hashimoto et al. (2008) employed distinctive muscle-like expandable cloth sleeves beneath the skin). Future comparative studies using varying types of androids would provide a better understanding of the relationship between structural differences and the displacement vector distribution.

## 5 Conclusion

We found two main facial deformation features that potentially characterized the human-likeness and were not observed in the androids: curved flow lines in the upper face and skin surface undulations where the upstream and downstream areas of the flow lines were elevated and depressed, respectively. In summary, the human facial motions were more complex than those of the androids. Innovative composite motion mechanisms to control both the flow lines and surface undulations are required to design advanced androids capable of exhibiting more realistic facial expressions. Measuring facial deformations in detail and using them to compare androids and humans is a promising approach for revealing current technology levels and identifying the inadequacy of state-of-the-art androids in a concrete and quantifiable manner. Further investigations with more numbers of humans will help us determine acceptable design variations for android faces and establish methods for designing androids with different personalities.

## Data Availability

The datasets generated for this study are available on request to the corresponding author.
